# Ribosomal protein L23 negatively regulates cellular apoptosis via the RPL23/Miz-1/c-Myc circuit in higher-risk myelodysplastic syndrome

**DOI:** 10.1038/s41598-017-02403-x

**Published:** 2017-05-24

**Authors:** Yuekun Qi, Xiao Li, Chunkang Chang, Feng Xu, Qi He, Youshan Zhao, Lingyun Wu

**Affiliations:** Department of Hematology, Shanghai Jiao Tong University Affiliated Sixth People’s Hospital, Shanghai, 200233 China

## Abstract

Ribosomal protein (RP) L23 is a negative regulator of cellular apoptosis, and RPL23 overexpression is associated with abnormal apoptotic resistance in CD34+ cells derived from patients with higher-risk myelodysplastic syndrome (MDS). However, the mechanism underlying RPL23-induced apoptotic resistance in higher-risk MDS patients is poorly understood. In this study, we showed that reduced RPL23 expression led to suppressed cellular viability, increased apoptosis and G1-S cell cycle arrest. Gene microarray analysis comparing RPL23-knockdown and control cells identified an array of differentially expressed genes, of which, Miz-1, was upregulated with transactivation of the cell cycle inhibitors p15^Ink4b^ and p21^Cip1^, and Miz-1’s functional repressor, c-Myc, was downregulated. Cells derived from higher-risk MDS patients demonstrated consistently increased expression of RPL23 and c-Myc and decreased Miz-1 expression compared with cells from lower-risk patients. In conclusion, Miz-1-dependent induction of p15^Ink4b^ and p21^Cip1^ was depressed with decreased Miz-1 and increased c-Myc expression under conditions of elevated RPL23 expression, leading to apoptotic resistance in higher-risk MDS patients. Because RPL23 is encoded by a target gene of c-Myc, the RPL23/Miz-1/c-Myc regulatory circuit provides a feedback loop that links efficient RPL23 expression with c-Myc’s function to suppress Miz-1-induced Cdk inhibitors and thereby leads to apoptotic resistance in higher-risk MDS patients.

## Introduction

Myelodysplastic syndrome (MDS) is a clonal haematopoietic stem cell disorder that is characterized by peripheral cytopenia, hypercellular bone marrow (BM), and increased mortality due to a substantial risk of progression to acute myeloid leukaemia (AML)^1^. Lower-risk (IPSS score ≤1.0) MDS patients primarily suffer from BM hypercellularity and peripheral cytopenia resulting from a significantly increased rate of apoptosis among BM haematopoietic cells. In contrast, BM haematopoietic cells in higher-risk (IPSS score >1.0) MDS patients demonstrate resistance to apoptosis, and some of these patients subsequently develop AML^2–4^. These opposing biological characteristics of BM haematopoietic cells lead to differences in disease progression and prognosis. However, the molecular pathogenesis underlying the apoptotic resistance observed in the BM haematopoietic cells of higher-risk MDS patients is poorly understood.

Ribosomal proteins (RPs), which are components of ribosomal subunits, are ubiquitous RNA-binding proteins that carry out multiple auxiliary extraribosomal functions and are moderately related to tumourigenesis^5–7^. Ribosomal protein L23 (RPL23) is a protein component of the 60S large ribosomal subunit. As previously reported, RPL23 acts as a negative regulator of apoptosis by suppressing Miz-1-induced transcriptional activation of the cell cycle inhibitors p15^Ink4b^ ^8^ and p21^Cip1^ ^9, 10^. Myc-associated zinc-finger protein (Miz-1) is a ubiquitous transcription factor that has been functionally characterized as a transcriptional inducer of p15^Ink4b^ and p21^Cip1^. However, in the presence of its repressor, c-Myc, the function of Miz-1 shifts from activation to transcriptional repression. c-Myc and Miz-1 form a co-repressor complex that silences Miz-1’s target genes, including the cell-cycle inhibitors p15^Ink4b^ and p21^Cip1^. Furthermore, Miz-1 quantitatively competes against and overcomes the inhibitory effects of c-Myc by competitively binding to promoters of its target genes^11, 12^.

A microarray analysis comprising 40,000 cDNA gene chip arrays conducted by Sridhar *et al*.^13^ identified 11 differentially expressed genes between CD34+ BM cells from tMDS (patients whose MDS transformed to AML) and sMDS (patients who remained stable). Overexpression of genes encoding five ribosomal proteins and other signalling pathways was observed in tMDS patients, and among these, RPL23 was overexpressed in tMDS patients compared with sMDS patients. Moreover, analyses of AML evolution and of the overexpressed genes in tMDS versus sMDS revealed that RPL23 overexpression was potentially involved in disease progression. A report from Li demonstrated significantly elevated RPL23 mRNA expression levels in higher-risk MDS patients, and these levels were inversely correlated with the percentage of apoptotic CD34+ cells, leading to disease progression and poor survival^14^.

This study further detected the RPL23 expression levels in MDS patients with different levels of risk and provided evidence supporting the anti-apoptotic effects of RPL23 in the BM cells of higher-risk MDS patients.

## Results

### RPL23 knockdown suppresses the viability of SKM-1 and K562 cells

**Figure 1:**
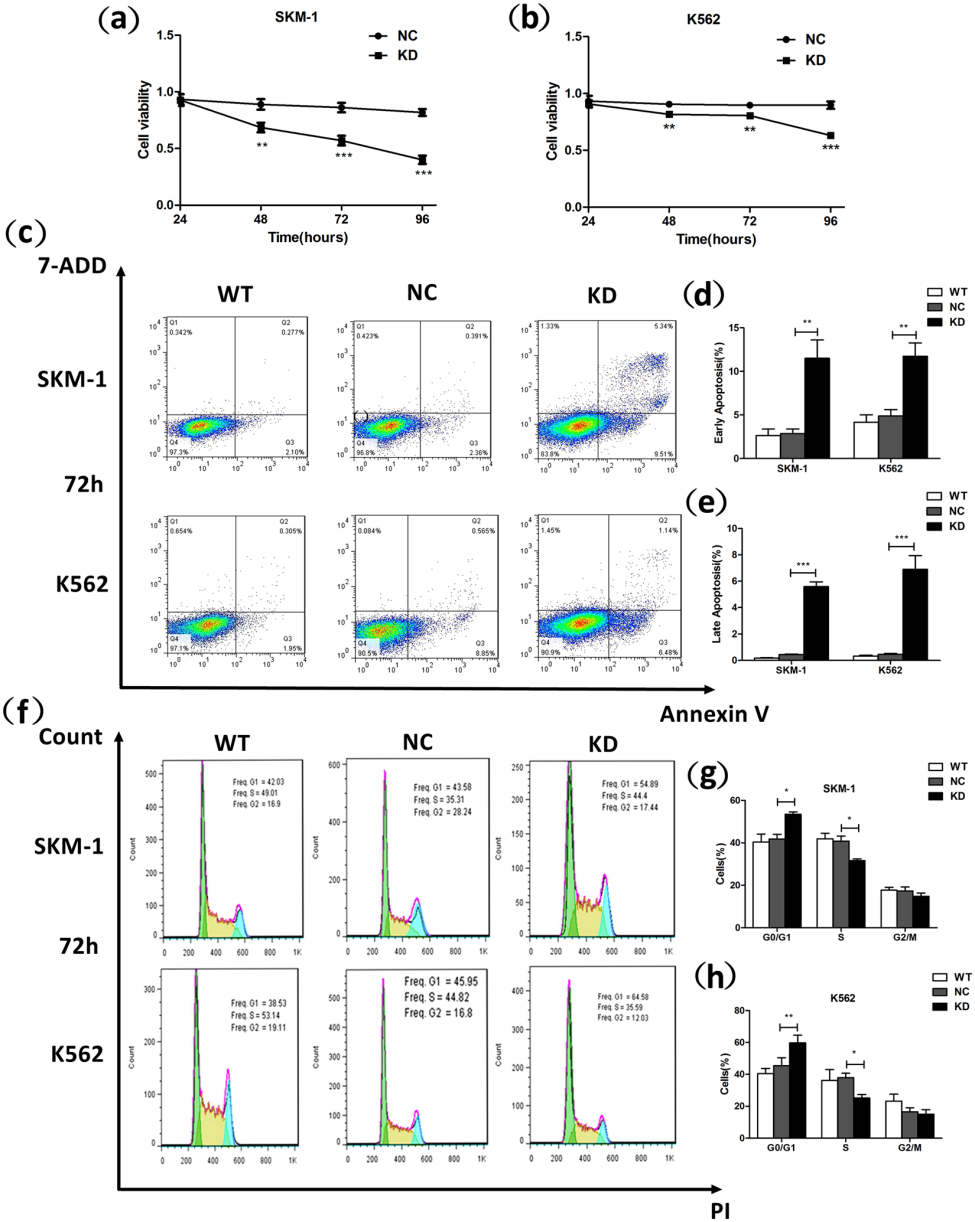
RPL23 knockdown blocks cell proliferation and induces apoptosis and cell cycle arrest. (**a**,**b**) The cell proliferation capacity of SKM-1/K562 cells was measured by a CCK-8 assay, and cellular viability was inhibited after RPL23 knockdown. (**c**) The representative flow graphic for apoptosis detection 72 h after efficient transfection of LV-RPL23-RNAi-GFP (KD group) and LV-RPL23-NC-GFP (NC group) in both SKM-1 and K562 cell lines is shown. The early-stage (**d**) and late-stage (**e**) apoptotic ratio increased after RPL23 knockdown. (**f**) The representative flow graphic for cell cycle detection 72 h after efficient transfection in both SKM-1 and K562 cell lines is shown. (**g**,**h**) The knockdown of RPL23 induced increases in the proportion of cells in the G0/G1 phase and a decreased proportion of cells in the S phase. Each assay was performed in triplicate. Unpaired Student’s t tests were used to calculate all *p* values shown in the figure. The data are expressed as the means ± S.E.M. WT: wild type; NC: RPL23-NC; KD: RPL23-KD. ^*^ *p* < 0.05; ^**^ *p* < 0.01; ^***^ *p* < 0.001.

We detected comparatively higher RPL23 mRNA expression in the SKM-1 [an acute myeloid leukaemia cell line established in the leukaemic phase during the progression from MDS to AML (MDS/AML)] and K562 [human chronic myelogenous leukaemia (CML) cell line] cell lines than in other leukaemic cell lines, e.g., HEL (human erythroleukaemia cell line), HL60 (human acute promyelocytic leukaemia cell line) and NB4 (human acute promyelocytic leukaemia cell line) (Supplementary Fig. S1a). Therefore, we selected the SKM-1 and K562 cell lines for subsequent biological function studies. To investigate the role of RPL23 in apoptotic-resistant, higher-risk MDS patients, the LV-RPL23-RNAi vector was designed and transfected into SKM-1 and K562 cells. The knockdown efficiency was verified by qRT-PCR (*p* < 0.001 for both cell lines; Supplementary Fig. S1b) and western blotting (*p* < 0.001 for SKM-1 and *p* = 0.005 for K562; Supplementary Fig. S1c and S1d) through comparison between the KD (vectors carrying LV-RPL23-RNAi-GFP) and NC (LV-RPL23-NC-GFP) groups. We measured the effects of RPL23-KD on cellular viability by performing CCK-8 assays (Fig. 1a and 1b). Twenty-four hours after efficient transfection, no changes were detected between the KD and NC groups (*p* > 0.05). However, the viability of SKM-1 cells decreased from 0.89 ± 0.02 to 0.69 ± 0.02 (*p* = 0.002) after 48 h, from 0.87 ± 0.02 to 0.57 ± 0.02 (*p* < 0.001) after 72 h, and from 0.82 ± 0.01 to 0.40 ± 0.02 (*p* < 0.001) after 96 h. Compared with the K562- NC group, the viability of K562-KD cells showed the following decreases: 48 h, 0.91 ± 0.01 vs. 0.82 ± 0.01 (*p* = 0.002); 72 h, 0.90 ± 0.01 vs. 0.81 ± 0.01 (*p* = 0.001); and 96 h, 0.89 ± 0.02 vs. 0.63 ± 0.01 (*p* < 0.001). Based on these results, the knockdown of RPL23 expression notably suppressed cellular viability in both cell lines.

### RPL23 knockdown increases apoptosis and induces cell cycle arrest of SKM1 and K562 cells

To investigate whether RPL23 knockdown interferes with cellular apoptosis, a flow cytometry analysis with Annexin V-APC/7-AAD double staining was performed. The results showed an increase in the number of early apoptotic cells of both cell lines compared with the NC group at different time points (SKM-1: 24 h, 4.75 ± 0.56% vs. 2.21 ± 0.44%, *p* = 0.037; 48 h, 7.06 ± 0.29% vs. 2.70 ± 0.47%, *p* = 0.016; K562: 24 h, 5.58 ± 0.36% vs. 3.81 ± 0.18%, *p* = 0.041; 48 h, 8.13 ± 0.43% vs. 4.66 ± 0.49%, *p* = 0.019). Seventy-two hours after efficient transfection, the early apoptotic ratio demonstrated substantial increases from 2.86 ± 0.31% to 11.50 ± 1.21% (*p* = 0.002) in SKM-1 cells and from 4.87 ± 0.42% to 11.74 ± 0.52% (*p* = 0.001) in K562 cells (Fig. 1c and 1d). Furthermore, the percentage of late-stage apoptotic cells was increased 72 h after transfection compared with the NC group (5.59 ± 0.36% vs. 0.44 ± 0.04% for SKM-1, *p* < 0.001; 6.90 ± 0.87% vs. 0.46 ± 0.08% for K562, *p* < 0.001; Fig. 1e). There was no difference in the apoptotic ratio between the NC group and the wild-type (WT) group. Pro-apoptotic induction due to RPL23 knockdown was evident based on a significant increase in a marker of apoptosis (cleaved caspase-3) in RPL23-KD SKM-1 cells, as detected by western blotting (Supplementary Fig. S1e, first panel). Cell cycle analysis revealed an increase in the proportion of cells in the G0/G1 phase 72 h after efficient transfection (SKM-1: 53.52 ± 1.86% vs. 40.39 ± 6.53%, *p* = 0.019; K562: 59.78 ± 4.85% vs. 45.52 ± 4.9%, *p* = 0.007) and a decreased proportion of cells in the S phase (SKM-1: 31.62 ± 1.48% vs. 40.84 ± 4.11%, *p* = 0.021; K562: 25.14 ± 2.25% vs. 37.93 ± 2.77%, *p* = 0.012), indicating cell cycle arrest at the G1-S checkpoint in both cell lines compared with NC cells (Fig. 1f–1h). No obvious difference in the cell cycle distribution was observed between the NC and WT cells.

### Global gene expression profiles

**Figure 2:**
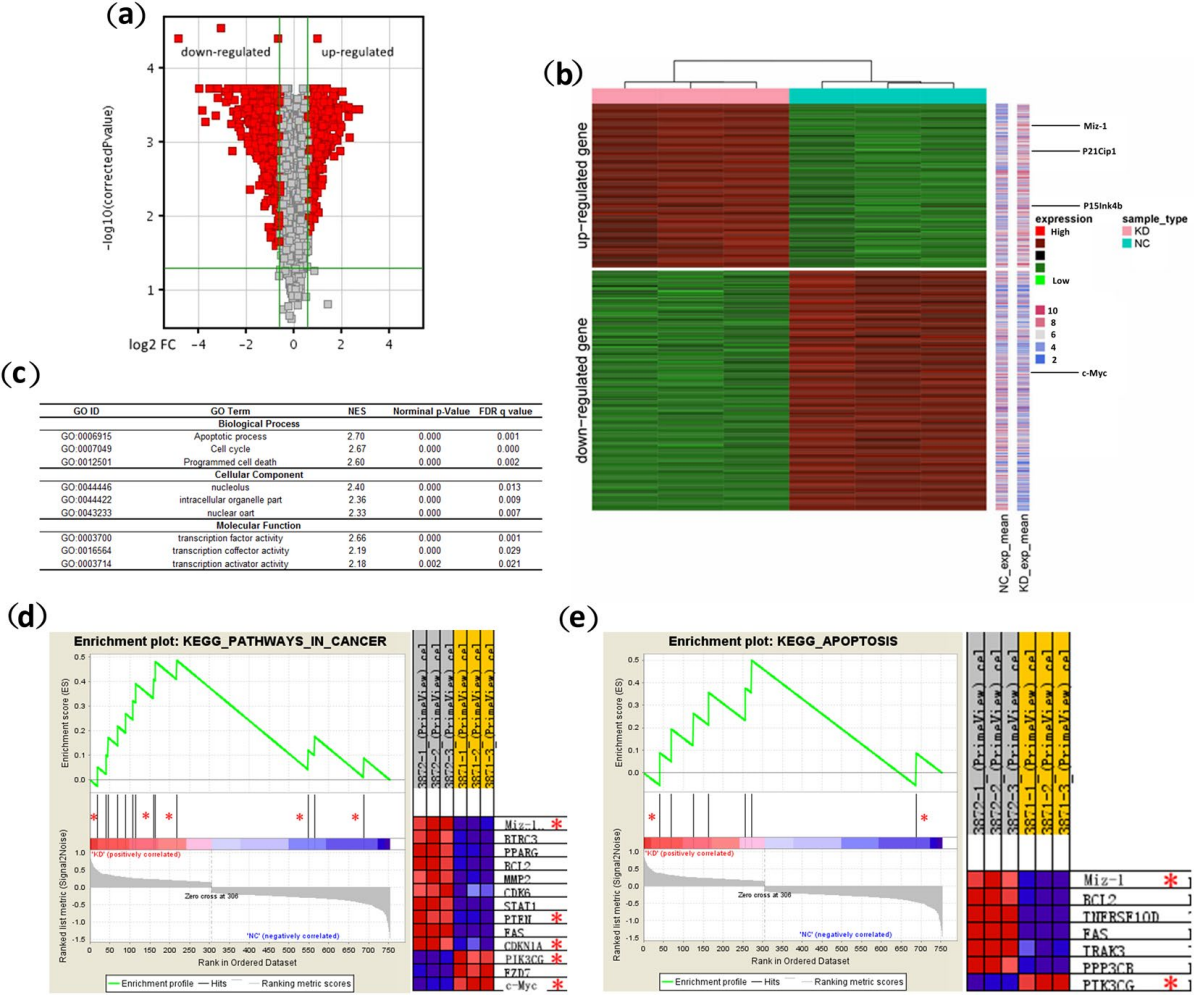
Global gene expression profiles and GSEA analysis. A DNA microarray analysis was performed using three RPL23-KD DNA samples and three NC DNA samples of infected SKM-1 cells. (**a**) A volcano plot of all assayed probes shows the distribution of DEGs based on the expression data from RPL23-KD and NC SKM-1 samples. The X-axis is the log_2_ (fold-change), and the Y-axis is the *p*-value on a −log_10_ scale. Entities with significant differences are highlighted in red. (**b**) Unsupervised hierarchical clustering of six samples shows distinct segregation based on treatment (pink row for RPL23-KD, blue row for NC SKM-1 cells). The expression of 753 probes was significantly altered (>1.5-fold) in RPL23-KD cells compared with NC samples (*p* < 0.05). A total of 305 probes (upper left region) were upregulated, and 448 (lower left region) were downregulated. Each row represents a gene, and each column represents a sample. The coloured bars on the right represent the absolute mean expression (absolute fold change) of each gene in the KD/NC groups. The indicated DEGs for our target molecules are shown on the right side of the bars. (**c**) Identified GO gene sets categorized by biological process, cellular component and molecular function. A GSEA analysis indicates the overrepresented pathways enriched in RPL23-KD SKM-1 cells: enrichment plots and heatmaps of leading edge genes in KEGG_PATHWAY IN CANCER (NES = 2.09) (**d**) and KEGG_APOTOSIS (NES = 1.65) (**e**). The asterisks mark DEGs involved in Miz-1/c-Myc function and PI3K/AKT signalling. GO ID/Term: Gene Ontology ID/term; NES: normalized enrichment score; FDR: false discovery rate.

To obtain insights into the molecular changes in response to RPL23 knockdown, the global changes in gene expression were examined by performing a DNA microarray analysis of RPL23-KD and RPL23-NC SKM-1 cells. As described in the Methods, a supervised unpaired T-test analysis using the Limma package produced a volcano plot depicting alterations in gene expression (Fig. 2a). Specifically, the results revealed 753 differentially expressed genes (DEGs), including 305 upregulated genes and 448 downregulated genes in the KD groups. The data from the gene expression arrays have been deposited in the NCBI Gene Expression Omnibus under GEO Accession Number GSE95348. A hierarchical clustering (HC) of the samples based on the transcript expression levels clearly segregated the transcripts into two distinct groups consistent with their treatments (Fig. 2b). Among the 753 DEGs, Miz-1 (FC = 6.53, *p* = 2.24E-06), CDKN1A (p21^Cip1^; FC = 2.98, *p* = 1.60E-05), and CDKN2B (p15^Ink4b^; FC = 2.57, *p* = 6.46E-05) were upregulated, whereas c-Myc (FC = −9.81, *p* = 2.62E-05) was downregulated. Additionally, PTEN (FC = 3.62, *p* = 3.87E-06) and PIK3CG (FC = −13.07, *p* = 8.62E-05) were notably differentially expressed in the RPL23-KD groups.

### Gene set enrichment analysis (GSEA)

We then explored whether the DEGs mapped to coherent functional gene sets and pathways. The array data were interpreted by performing a GSEA analysis through annotation with the C2: Kyoto Encyclopedia of Genes and Genomics (KEGG) pathways and C5: Gene Ontology (GO) gene sets. The DEGs were assigned to multiple C5: GO gene sets categorized by biological process, cellular component and molecular function^15–17^. The major biological processes associated with these DEGs were apoptotic processes, cell cycle and programmed cell death, consistent with the growth inhibition observed during our functional experiments. The following cellular component gene sets were enriched in RPL23-KD cells: nucleolus, intracellular organelles and nucleus, covering genes associated with ribosomal biogenesis within the nucleus as well as nuclear stress^5^. The enriched molecular function gene sets mainly involved transcriptional regulation. Detailed information is provided in Fig. 2c. Pathway annotation based on the KEGG database indicated that pathways in cancer (NES = 2.09, nominal *p* < 0.001, FDR *q* = 0.038) and apoptosis (NES = 1.65, nominal *p* = 0.033, FDR *q* = 0.199) were highly enriched, and the leading-edge subsets included Miz-1, CDKN1A (p21^Cip1^), c-Myc, PIK3CG, PTEN and other apoptosis-related genes (e.g., BCL2, FAS, and CDK6; Fig. 2d and 2e).

### Validation of screened mRNAs identified from the microarray analysis through qRT-PCR and western blotting of SKM-1 cells

**Figure 3:**
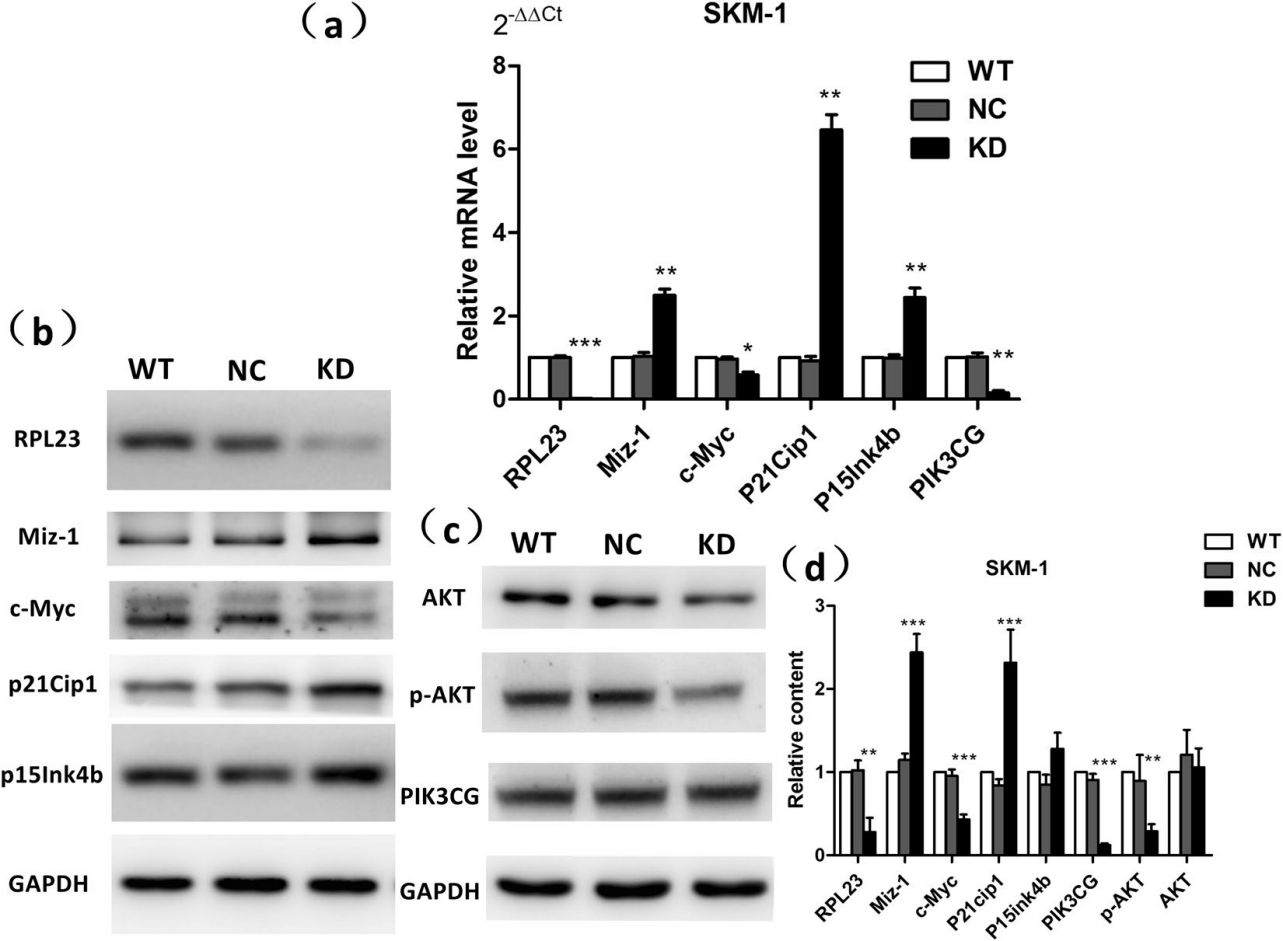
Validation of screened mRNAs identified by microarray analysis through qRT-PCR and western blotting of SKM-1 cells. (**a**) The mRNA expression levels of RPL23-KD-associated genes were analysed by qRT-PCR. The Miz-1 (2.50 ± 0.14, *p* = 0.009), p15^Ink4b^ (2.44 ± 0.23, *p* = 0.003), and p21^Cip1^ (6.46 ± 0.37, *p* = 0.005) expression levels were increased in the KD group compared with the NC group, whereas the RPL23 (0.02 ± 0.01, *p* < 0.001), c-Myc (0.58 ± 0.08, *p* = 0.031) and PI3KCG (0.15 ± 0.05, *p* = 0.004) expression levels were decreased with RPL23 knockdown. (**b**,**c**) The western blotting results revealed altered protein levels, specifically increased expression of Miz-1 (*p* < 0.001) and its downstream target molecules, p21^Cip1^ (*p* < 0.001) and p15^Ink4b^ (*p* = 0.09), and decreased expression of c-Myc (*p* < 0.001) and PIK3CG (*p* < 0.001). The phosphorylation of AKT was also weakened (*p* = 0.008). (**d**) Histogram denoting the relative grey scale of the target stripes normalized to the GAPDH expression level using Image-Pro Plus v6.0 software (Media Cybernetics, USA). Target stripes were displayed as chopped. Original scans were provided in Supplementary Information (Supplementary Fig. S5). Each assay was performed in triplicate. Unpaired Student’s t tests were used to calculate all *p* values shown throughout the figure. The data are expressed as the means ± S.E.M. WT: wild type; NC: RPL23-NC; KD: RPL23-KD. ^*^ *p* < 0.05; ^**^ *p* < 0.01; ^***^ *p* < 0.001.

We performed qRT-PCR and western blotting analyses of RPL23-KD/NC SKM-1 cells to verify the expression profiles obtained in the microarray analysis and to further investigate changes in the related protein levels. The relative mRNA expression levels are shown in Fig. 3a: RPL23 (0.02 ± 0.01, *p* < 0.001), Miz-1 (2.50 ± 0.14, *p* = 0.009), c-Myc (0.58 ± 0.08, *p* = 0.031), p15^Ink4b^ (2.44 ± 0.23, *p* = 0.003), p21^Cip1^ (6.46 ± 0.37, *p* = 0.005), and PI3KCG (0.15 ± 0.05, *p* = 0.004). The mRNA expression levels of the RPL23-KD/NC groups were normalized to those of the WT group. p15^Ink4b^ and p21^Cip1^ were transcriptionally activated following RPL23 knockdown, and this effect was accompanied by increased Miz-1 and decreased c-Myc expression. Our western blotting results also revealed altered protein levels, specifically increased expression of Miz-1 (*p* < 0.001) and its downstream target molecules, p21^Cip1^ (*p* < 0.001) and p15^Ink4b^ (which exhibited a mild trend: *p* = 0.09) and decreased expression of c-Myc (*p* < 0.001) and PIK3CG (*p* < 0.001), as shown in Fig. 3b and 3c. The phosphorylation of AKT, a downstream effector of PI3K/AKT signalling, was also weakened (*p* = 0.008), indicating suppression of PI3K/AKT signalling activity.

### Expression of c-Myc overcomes RPL23-KD-induced viability suppression and apoptotic tendency

**Figure 4:**
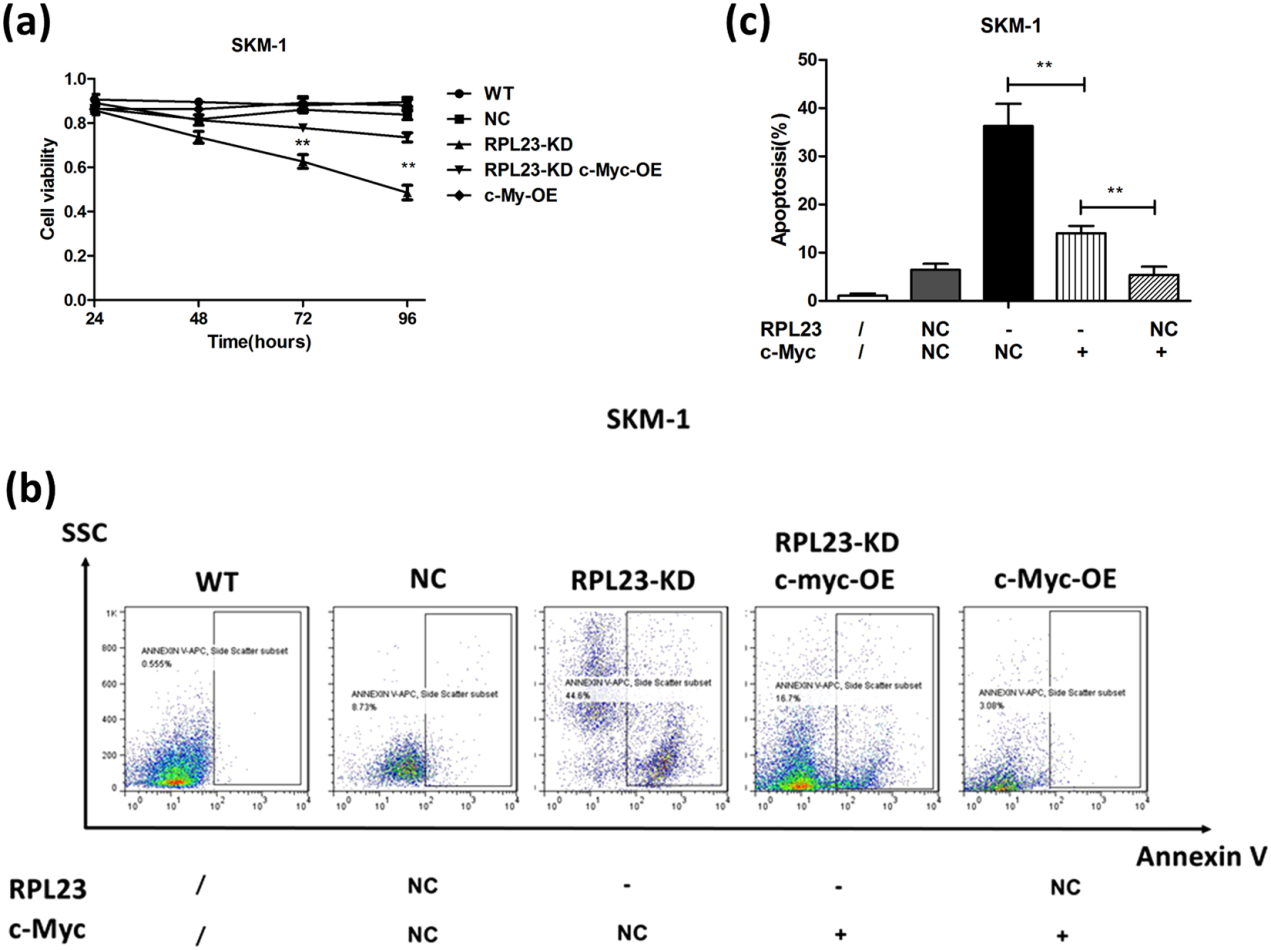
Expression of c-Myc overcomes RPL23-KD-induced viability suppression and apoptotic tendency in SKM-1 cells. (**a**) Growth curve of SKM-1 cells infected with LV-RPL23-RNAi expression vectors alone or together with CMV–c-Myc expression plasmid vectors after superinfection at the indicated time point. Suppressive proliferation of RPL23-KD cells was overcome by CMV-driven expression of c-Myc (p < 0.01). (**b**,**c**) Forty-eight hours after superinfection, cellular apoptosis was measured by Annexin V single-staining, and induced expression of c-Myc alleviated the high apoptotic potential that is mediated by depletion of L23. Unpaired Student’s t tests were used to calculate all *p* values shown throughout the figure. The data are expressed as the means ± S.E.M. WT: non-transfected group (used as blank control); NC: superinfected with two empty vectors (used as negative control); RPL23-KD: superinfected with LV-RPL23- RNAi and CMV-c-Myc-NC; RPL23-KD/c-Myc-OE: superinfected with LV-RPL23- RNAi and CMV-c-Myc and c-Myc-OE: superinfected with CMV-c-Myc and LV-RPL23-NC. /: non-treated; −: decreased expression; +: induced expression; NC: transfected with empty vectors. ^*^ *p* < 0.05; ^**^ *p* < 0.01; ^***^ *p* < 0.001.

To determine whether c-Myc plays an important role in RPL23-knockdown-mediated biological alterations and whether the detected changes in c-Myc expression are associated with RPL23 knockdown, SKM-1 cells were first transfected with LV-RPL23-RNAi, and twenty-four hours later, the transfected SKM-1 cells were superinfected with CMV-driven expression plasmids encoding c-Myc (CMV-c-Myc). Due to the vulnerable cellular characteristics of RPL23-KD SKM-1 cells, we used a multiplicity of infection (MOI) of 10 for LV-RPL23-RNAi transfection without fluorescence-activated cell sorting (FACS). Detailed information of the superinfection is provided in the Supplementary Information. The efficiency of superinfection with LV-RPL23-RNAi and CMV-c-Myc was measured by qRT-PCR (Supplementary Fig. S2). We performed cellular proliferation and apoptosis analyses of the following 5 groups: (a) WT, non-transfected group (used as blank control); (b) NC, superinfected with two empty vectors (used as negative control); (c) RPL23-KD, superinfected with LV-RPL23-RNAi and CMV-c-Myc-NC; (d) RPL23-KD/c-Myc-OE, superinfected with LV-RPL23-RNAi and CMV-c-Myc; and (e) c-Myc-OE, superinfected with CMV-c-Myc and LV-RPL23-NC. The cellular viability was measured twenty-four hours after coinfection using CCK-8 (Fig. 4a). The growth suppressive effect of RPL23-KD was overcome by CMV-driven expression of c-Myc (*p* < 0.01). Note that c-Myc overexpression alone resulted in a higher proliferative ratio than that of the RPL23-KD-c-Myc-OE group (*p* < 0.05). Forty-eight hours after superinfection, cellular apoptosis was measured by Annexin V single-staining of specific cells (Fig. 4b and 4c﻿), and induced expression of c-Myc alleviated the high apoptotic potential mediated by depletion of L23 (from 36.33 ± 4.55% to 14.07 ± 1.50%, *p* = 0.002). Furthermore, the induced expression of c-Myc alone in SKM-1 cells displayed a lower apoptotic ratio (5.42 ± 1.68%, *p* = 0.003) in the absence of LV-RPL23-RNAi. Detailed information of the gating protocol used for the flow cytometry data is described in Supplementary Fig. S3.

### Expression of RPL23, Miz-1 and c-Myc in the BM haematopoietic cells of MDS patients

**Figure 5:**
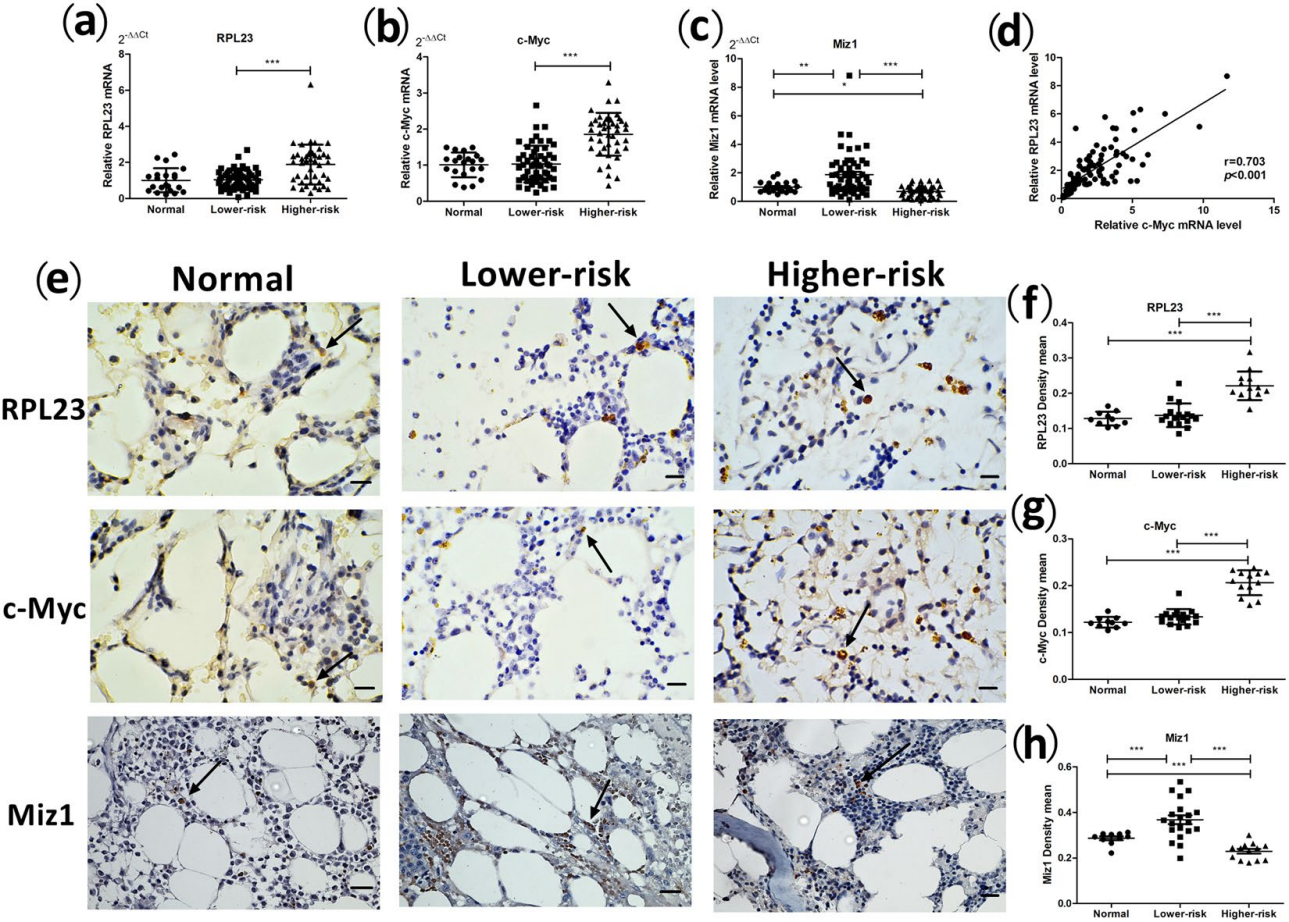
RPL23, c-Myc and Miz1 mRNA and protein expression levels in the BM haematopoietic cells of MDS patients. (**a**) RPL23, (**b**) c-Myc and (**c**) Miz-1 mRNA expression levels in lower-risk (IPSS scores ≤1.0) and higher-risk (IPSS scores >1.0) MDS patients compared with healthy donors. Total RNA was extracted from 2 ml of BM mononuclear cells (BMNCs) from MDS patients with different risk levels using the TRIzol® reagent. The protocols used for cDNA synthesis and qRT-PCR are described in Methods. (**d**) Correlation between RPL23 and c-Myc mRNA levels in BMNC cells from MDS patients. Pearson’s correlation test for RPL23 vs. c-Myc: *r* = 0.703, *p* < 0.000. (**e**) Immunohistochemical staining for RPL23, c-Myc and Miz-1 in BM trephine tissue of lower/higher-risk MDS patients and healthy donors. RPL23, c-Myc and Miz-1 expression is indicated by yellowish-brown cytoplasmic staining. Original magnification: 400×. Clearly positively stained cells are indicated with arrows (scale bar: 2 μm). The mean densities of the positive sections were calculated for measuring *in vivo* protein expression and are displayed in scatter plots in panels (**f**), (**g**) and (**h**). Unpaired Student’s t tests were used to calculate all *p* values shown throughout the figure. The data are expressed as the means ± S.E.M. ^*^ *p* < 0.05; ^**^ *p* < 0.01; ^***^ *p* < 0.001.

To investigate the expression levels of RPL23 and the related molecules Miz-1 and c-Myc in the BM haematopoietic cells of MDS patients, qRT-PCR and immunohistochemical (IHC) analyses of samples from MDS patients with different levels of risk were performed (the patients’ information is listed in Supplementary Table S1). The RPL23, Miz-1 and c-Myc mRNA expression levels, which were calculated using the 2^−△△Ct^ method (mean ± S.E.M), were evaluated in 97 MDS samples (54 lower-risk and 43 higher-risk MDS patients) and compared with the levels in corresponding samples from 21 normal donors (Fig. 5a–5c). The RPL23 expression levels in the lower- and higher-risk MDS patients were 1.05 ± 0.07 and 1.88 ± 0.17, respectively (*p* < 0.001), and the c-Myc expression levels were 1.03 ± 0.07 and 1.86 ± 0.09 in the lower- and higher-risk MDS patients, respectively (*p* < 0.001). Miz-1 demonstrated higher expression in lower-risk MDS patients compared with higher-risk patients (1.88 ± 0.20 vs. 0.69 ± 0.06, *p* < 0.001) and normal controls (*p* = 0.001). We also observed lower Miz-1 expression in higher-risk patients than in normal donors (*p* = 0.035). A Pearson’s correlation analysis was performed to analyse the RPL23 and c-Myc mRNA levels (Fig. 5d), and RPL23 expression was found to be positively correlated with c-Myc expression (*r* = 0.703, *p* < 0.001). Additionally, a relatively weak negative correlation was found between the RPL23 and Miz-1 mRNA levels (*r* = −0.25, *p* = 0.01).

An IHC analysis of target protein expression was performed by determining the mean density of positive staining. The mean densities for RPL23 in BM haematopoietic cells from lower- and higher-risk MDS patients were 0.14 ± 0.01 and 0.21 ± 0.01 (*p* < 0.001), whereas the normal donors exhibited no difference compared with lower-risk patients (0.13 ± 0.01, *p* = 0.44). The mean densities for c-Myc in the cells from lower- and higher-risk MDS patients were 0.13 ± 0.00 vs. 0.21 ± 0.01, respectively (*p* < 0.001), whereas that obtained for the cells from the normal donors was 0.12 ± 0.00. The expression intensity of Miz-1 was 0.37 ± 0.02 in lower-risk MDS patients, whereas higher-risk MDS patients exhibited decreased Miz-1 expression (0.23 ± 0.01, *p* < 0.001), and this decreased expression was even lower than that observed in normal donors (0.29 ± 0.01, *p* < 0.001; Fig. 5e–5h).

## Discussion

RPL23 is a protein component of the 60S large ribosomal subunit and is believed to perform multiple auxiliary extraribosomal functions; additionally, RPL23 negatively regulates apoptosis by suppressing the Miz-1-induced transcription of the cell cycle inhibitors p15^Ink4b^ and p21^Cip1^. As reported before, RPL23 is overexpressed at the mRNA level in higher-risk MDS patients, and elevated RPL23 expression is inversely associated with the apoptosis ratio in CD34+ BM cells, which might lead to disease progression and adverse prognosis. However, the mechanism underlying this effect remains uncharacterized.

In this study, knockdown of RPL23 was found to suppress SKM-1/K562 cellular viability by strongly inducing apoptotic cell death and G1/S phase arrest. To further investigate the molecular mechanisms involved in these phenomena, we performed a gene microarray analysis of RPL23-KD and RPL23-NC samples and detected elevated expression of Miz-1 and its target molecules p15^Ink4b^ and p21^Cip1^ as well as decreased expression of c-Myc. To further elucidate the potential regulatory mechanisms involving c-Myc/Miz-1, a rescue experiment using SKM-1 cells co-infected with LV-RPL23-RNAi and CMV-c-Myc-expressing plasmid vectors was performed, and the results demonstrated that the induction of c-Myc expression could overcome the higher tendency to apoptosis mediated by RPL23-KD, supporting the conclusion that c-Myc is an important downstream regulator of RPL23-mediated abnormalities in apoptosis. The analysis of the patients’ data confirmed this deregulation and correlated the RPL23/Miz-1/c-Myc expression levels with the risk levels of MDS patients.

Compared with patients presenting normal RPL23 expression levels, higher-risk MDS patients with increased RPL23 expression demonstrated decreased Miz-1 expression, which resulted in suppression of Miz-1-induced p15^Ink4b^ and p21^Cip1^ expression in their BM haematopoietic cells. p15^Ink4b^ and p21^Cip1^ downregulation promotes cell cycle progression in the BM haematopoietic cells of MDS and myeloid disease patients and transforms their tendency to undergo apoptosis into apoptosis resistance^18–20^. Consistently, lower-risk MDS patients exhibited elevated Miz-1 expression compared with normal donors, which potentially accounted for the BM haematopoietic cells’ tendency toward apoptosis observed in lower-risk MDS patients. Additionally, elevated c-Myc expression was also observed in higher-risk MDS patients, and *in vitro* experiments and microarray analyses indicated that RPL23 knockdown was potentially associated with decreased c-Myc expression as well as suppressed PI3K/AKT signalling. According to a previous study, PI3K/AKT activity is positively associated with c-Myc regulation. Phosphorylated AKT leads to the transcriptional and post-translational induction of c-Myc^21–23^. Our observation of depressed PI3K/AKT activity accompanied by decreased c-Myc expression in RPL23-KD cells precisely corresponds with this regulation mode, and the observed c-Myc downregulation, which might be associated with the repressed PI3K/AKT activity after RPL23 knockdown, might attenuated the Myc-Miz-1 interaction and therefore enhanced Miz-1 function. In MDS patients, the upregulation of c-Myc and the suppression of Miz-1 activity synergistically lead to apoptotic resistance in higher-risk MDS patients with elevated RPL23 expression.

**Figure 6:**
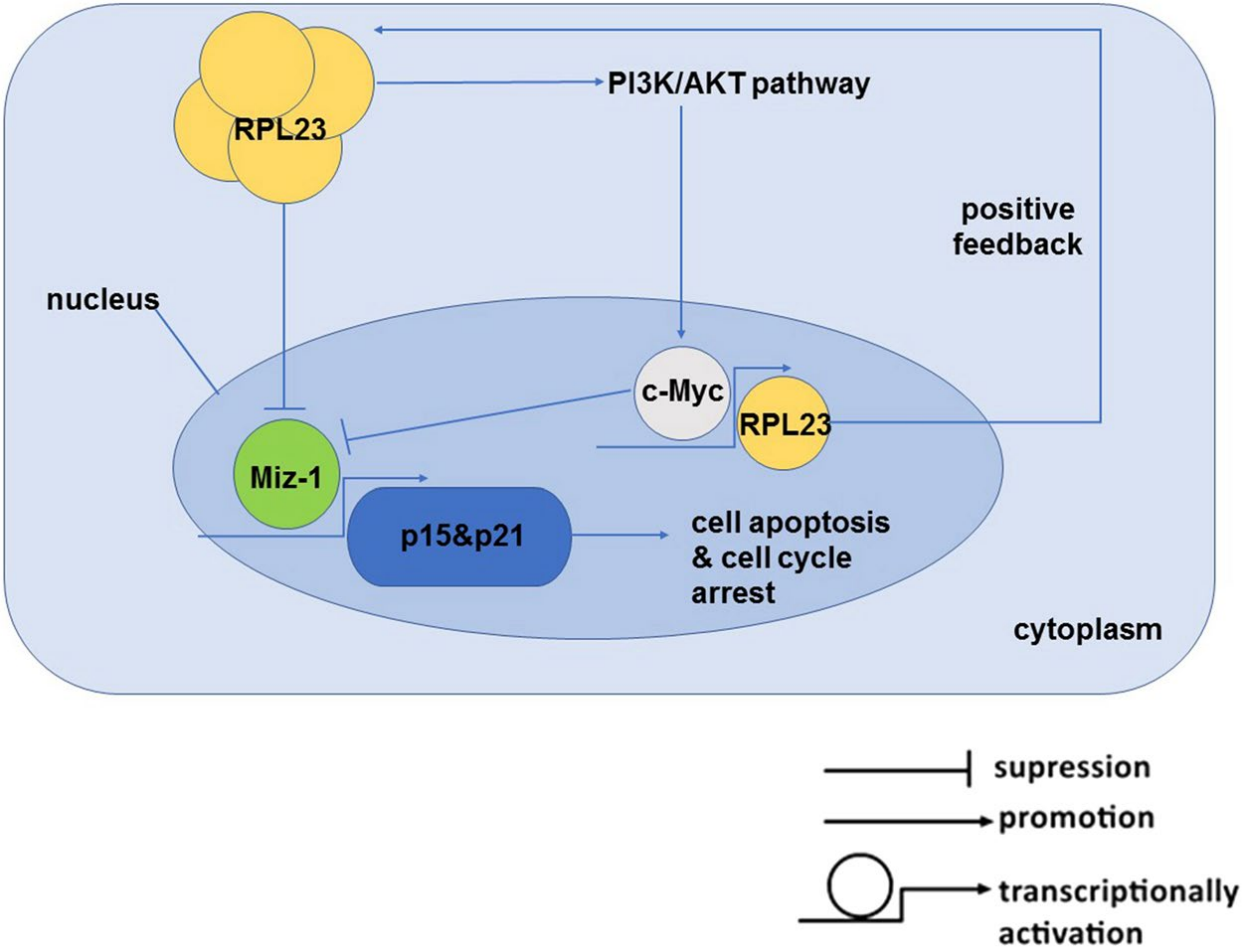
Schematic showing the potential role of RPL23 in the RPL23/Miz-1/c-Myc circuit. Our study suggests a regulatory feedback loop through which RPL23 reinforces the Myc-dependent oncogenic functions. Elevated expression of RPL23 would decrease the Miz-1-dependent expression of p21^Cip1^ and p15^Ink4b^, thereby increasing the ability of c-Myc to promote cell cycle progression. In turn, the activation of c-Myc function in the context of RPL23 overexpression might further stimulate RPL23 expression, representing a positive feedback mechanism that couples efficient RPL23 expression with c-Myc’s function to suppress Miz-1-induced Cdk inhibitors and thereby promote cell cycle progression and induce apoptotic resistance.

RPL23, which is encoded by a gene that is upregulated by c-Myc, has an essential function in restricting Miz-1-dependent cell-cycle arrest^24^. Our study suggests a regulatory feedback loop in which RPL23 could reinforce c-Myc-dependent oncogenic functions. Elevated expression of RPL23 could decrease the Miz-1-dependent expression of p21^Cip1^ and p15^Ink4b^, thereby increasing the ability of c-Myc to promote cell-cycle progression. In turn, the activation of c-Myc function in the context of RPL23 overexpression might further stimulate RPL23 expression. Thus, the regulation of Miz-1 activity by RPL23 represents a positive feedback mechanism that couples efficient RPL23 expression with the function of c-Myc to suppress Miz-1-induced Cdk inhibitors and thereby promote cell-cycle progression (Fig. 6). In higher-risk MDS, RPL23 overexpression might confer growth advantages and resistance to apoptosis through this positive feedback loop, potentially leading to AML evolution.

According to our functional experiments, the knockdown of RPL23 expression in SKM-1 and K562 cells changed the cellular proliferation rate, the apoptotic ratio and the cell cycle distribution. Considering the known roles of Miz-1 and c-Myc in cellular senescence, we may wonder whether the RPL23-KD cells exhibit altered cellular senescence. The β-galactosidase staining of RPL23-NC/KD and wild-type SKM-1/K562 cells revealed that the percentages of SA-gal-positive cells (senescent signals) did not show any significant differences among the three groups in both cell lines (Supplementary Fig. S4). The following explanations might account for the undetected senescent signals observed in our study. (1) The SKM-1 and K562 cell lines, which were subjected to RPL23 knockdown and senescence analysis, are both haematological cancer cell lines. Cancer cells show the characteristics of immortalization and absence of senescence; thus, these two cell lines, including the RPL23-knockdown and blank/negative control groups, barely showed any senescent signals. (2) MDS is a clonal haematopoietic stem cell disorder that is characterized by bone marrow cell dysplasia and ineffective haematopoiesis^1^. Cellular senescence is a feature of this disease (e.g., observed in BM mesenchymal stromal cells (MSCs) of MDS patients) but is not the dominant feature of haematopoietic cells, particularly in patients whose disease has evolved to AML or in patients with chromosomal instability (e.g., patients with an isolated monosomy 7 showed significantly longer telomeres and weak senescent signals^25, 26^). It remains to be investigated whether cellular senescence might be involved in the potential mechanisms resulting in RPL23 overexpression MDS patients or is a subordinate aspect. Interestingly, RPL23 has been reported to functionally inhibit the HDM2 ubiquitin ligase and thereby activate p53, leading to growth inhibition and anti-tumour effects in cases of gastric cancer^27–29^. In our RPL23-KD SKM-1 cells, however, decreased RPL23 expression was correlated with the induction of apoptosis, and our western blotting results did not reveal any alternations in p53 expression. Furthermore, the downstream target gene of p53, HDM2, was not transcriptionally activated, as demonstrated by our qRT-PCR analysis (Supplementary Fig. S1e second panel and S1f). To the best of our knowledge, the SKM-1 cell line, which was established in the leukaemic phase during the progression from MDS to AML, is karyotypically abnormal (including a 17p deletion) and is characteristic of the acquisition of p53 mutations (missense and silent point mutations)^30, 31^. Mutation of the p53 gene is associated with complex karyotypes, reduces overall survival and plays a role in the evolution of MDS to AML^30–32^. Therefore, the loss of normal p53 gene function, i.e., the allelic inactivation of 17p and the point mutation of the other allelic p53 gene in the SKM-1 cell line, might give rise to undetected p53 alterations and RPL23-HDM2-p53 signal activation, as previously reported. Regarding the clinical cases of elevated RPL23 expression in higher-risk MDS patients, the two opposing roles of RPL23 in cellular apoptosis might be the result of heterogeneity in individual cells and disease stages in different MDS patients, an effect that is complicated by different karyotype abnormalities and p53 mutation scenarios. Furthermore, the RPL23 expression levels of our patients presented significant variation among higher-risk MDS patients, and some patients with a higher-risk phenotype exhibited normal or lower RPL23 expression levels. However, the overall expression trend was higher among higher-risk MDS patients than among lower-risk MDS patients and normal donors. As reported previously, DBA and 5q- MDS are associated with inherited (DBA) or acquired (5q- MDS) ribosomal protein haploinsufficiency through ribosomal protein gene mutations or karyotype abnormalities (e.g., chromosome loss)^33, 34^. Therefore, the heterogeneity of RPL23 expression levels in MDS patients might result from the haploinsufficiency of RPL23 protein production in patients with different related chromosomal abnormalities and complex gene mutations.

In summary, RPL23 expression levels have been identified as an independent predictor of prognosis ﻿of MDS patients, regardless of patients’ age, IPSS score, or haemoglobin levels^14^. Based on these findings, RPL23 dysregulation involves a novel molecular mechanism in MDS pathogenesis and represents a potential prognostic biomarker and novel therapeutic target in patients with MDS.

## Methods

### Patients and control samples

All 97 patients enrolled in this study were diagnosed with MDS by our department according to the WHO 2008 classification criteria and the minimal diagnostic standards for MDS. Following approval by the local ethics committee, BM samples were obtained upon MDS diagnosis prior to the initiation of any treatment. Each of the 97 MDS patients and 21 normal donors provided written informed consent. For risk level classification, 54 patients with international prognostic scoring system (IPSS) scores of at most 1.0 were included in the lower-risk MDS group (nine low- and 45 int-1-risk patients), whereas 43 MDS patients with IPSS scores greater than 1.0 were included in the higher-risk MDS group (33 int-2- and 10 high-risk patients). The characteristics of the MDS patients are listed in Supplementary Table S1. The research was approved by the ethics committee of Shanghai Jiao Tong University Affiliated Sixth People’s Hospital, and all clinical information was obtained in accordance with approved guidelines based on the Declaration of Helsinki. All study participants signed an informed consent form.

### Cell lines and reagents

SKM-1 [an acute myeloid leukaemia cell line established in the leukaemic phase during the progression from MDS to AML (MDS/AML)] and K562 [human chronic myelogenous leukaemia (CML) cell line] cells (Health Science Research Resources Bank, Japan) were grown in RPMI-1640 medium (Gibco, US) containing 10% heat-inactivated foetal bovine serum, 100 IU/ml penicillin, and 100 μg/ml streptomycin in a humidified atmosphere of 5% CO_2_ at 37 °C.

### Preparation of lentiviral RNAi vectors and expression plasmid vectors and cell infection

An RNA interference fragment was designed based on the RPL23 sequence (GenBank No.: NM_000978.3), and the effective target genes of RPL23 RNAi were screened. Oligonucleotides of the target sequences were synthesized and packed into lentiviral vectors (pGCSIL-GFP) to generate lentiviral particles from Genechem, Inc. (Shanghai, China). Transfection of SKM-1 and K562 cells with LV-RPL23-RNAi/NC was performed using 5 mg/ml Polybrene (Genechem, Shanghai, China) at an appropriate MOI of 20 according to the manufacturer’s instructions, and the medium was changed 12 h after transfection. For the induction of c-Myc expression in SKM-1 cells, recombinant plasmid vectors encoding c-Myc (GenBank No. NM_002467) were generated using pcDNA3.1-RFP plasmids (Biovector, Inc., USA), and expression induction was confirmed by PCR, restriction analysis and DNA sequencing (Biovector, Inc.). Transient transfection of CMV-c-Myc-RFP plasmid vectors was performed using Lipofectamine® 2000 Transfection Reagent (Invitrogen) according to the manufacturer’s instructions. The following RNAi sequences were used to generate lentiviral vectors for RPL23 depletion: RPL23-RNAi1, 5′-GAT CCC CAG TGG TCA TTC GAC AAC GAT TCA AGA GAT CGT TGT CGA ATG ACC ACT TTT TTG GAA A-3′; and RPL23-RNAi2, 5′-GAT CCC CAG ATG GCG TGT TTC TTT ATT TCA AGA GAA TAA AGA AAC ACG CCA TCT TTT TTG GAA A-3′. The following primers were used for the *in vitro* amplification of c-Myc: F, 5′-CCG GAA TTC ATG GAT TTT TTTCGG GTA-3′; and R, 5′-TCC CCC GGG TTA CGC ACA AGA GTT CCG TA-3′. Wild type SKM-1 and K562 cells were used as blank controls (WT group).

### Cellular functional assays

The cellular viability was measured by performing a CCK-8 assay (Dojindo, kamimashiki gun Kumamoto, Japan) following the manufacturer’s instructions. Briefly, SKM-1 and K562 cells infected with LV-RPL23-RNAi or LV-NC, as well as untreated cells, were seeded into 96-well plates (4,000 cells/well) in a volume of 100 μl. Each experiment was performed with four replicates. After 24, 48, 72, and 96 h, 10 μl of CCK-8 solution was added to each well, and the plate was incubated at 37 °C for 2–4 h. The absorbance at 450 nm was measured with a microplate reader (Biotech, NY, USA). The percentage of viable cells was calculated using the following formula: cell viability = [OD (Treatment) − OD (Blank)]/[OD (Control) − OD (Blank)]. The results were normalized against the blank control without lentiviral transfection.

Cellular apoptosis was evaluated using an Annexin V-APC/7-AAD Apoptosis Kit (MultiSciences, Hangzhou, China) following the manufacturer’s instructions. SKM-1 and K562 cells were seeded into six-well plates (1 × 10^5^/well) and infected as previously mentioned. After 24, 48, and 72 h, the cells were harvested for FCM analysis (Calibur, BD Biosciences, CA, USA). For assessment of the cell cycle distribution, the cells were harvested 72 h after infection, washed twice with ice-cold PBS and fixed overnight at 4 °C in ice-cold 70% ethanol. The fixed cells were resuspended in PBS containing 50 μg/ml RNase A (Invitrogen, Carlsbad, CA, USA) and 50 μg/ml propidium iodide (Sigma, St. Louis, MO, USA) and incubated at 37 °C in the dark for 1 h prior to FCM analysis. The data were interpreted using FlowJo software (version 3.2, Verity Software House, USA).

### RNA isolation and qRT-PCR

SKM-1 cells were harvested three days after lentiviral infection. Total RNA was extracted using the TRIzol® reagent (Invitrogen) and was reverse-transcribed into cDNA using a SuperRT cDNA kit (CWBIO, Beijing, China) according to the manufacturer’s instructions. Total patient RNA was extracted from 2 ml of BM mononuclear cells (BMNCs) and reverse-transcribed into cDNA. PCR reactions for RPL23 and other target genes were performed using SYBR Green Master Mix (Takara, Dalian, China) and an ABI PRISM 7500 System (Applied Biosystems, Foster, CA, USA). The relative expression of the target genes was normalized to the β-actin expression levels and was calculated using the 2^−ΔΔCt^ method. The detailed primer sequences are listed in Supplementary Table S2.

### Microarray hybridization and analysis

The total RNA from RPL23-KD and NC SKM-1 cells (triplicate per treatment category) was isolated as described above. cDNA and biotinylated cRNA were synthesized and hybridized to a GeneChip® PrimeView™ Human Gene Expression Array (Affymetrix, Santa Clara, CA, USA) according to the manufacturer’ instructions. Background correction, normalization, and expression calculations were performed using Robust Multichip Average (RMA). Probe IDs were annotated based on the annotation database from NCBI-GEO (Platform No. GPL15207) to generate a gene symbol list. The gene list was filtered with a fold change cut-off of 1.5 and *p* < 0.05 by performing a supervised unpaired T-test analysis with the Limma package in R Bioconductor.

### Gene set enrichment analysis (GSEA)

Gene Set Enrichment Analysis (GSEA) was performed using GSEA v2.2.0 (Broad Institute, Cambridge, USA) to obtain gene sets that demonstrated statistically significant differences in expression between the NC and KD groups. The C2: Kyoto Encyclopedia of Genes and Genomics (KEGG) gene set and the C5: Gene Ontology (GO) gene set were used for gene set annotation. The thresholds for the nominal *p*-value and FDR *q*-value were set to 0.01 and 0.25, respectively.

### Western blot analysis

Following treatment, the cells were lysed with RIPA buffer (Beyotime, China) in the presence of a protease inhibitor cocktail and a phosphatase inhibitor cocktail (Biotool, USA), both at a dilution of 1:100 (v/v). The protein concentrations were determined using a BCA protein assay kit (CWBIO, China).

Fifty micrograms of proteins were separated by SDS-PAGE and transferred to PVDF membranes (Merck Millipore, Germany). After incubation in 5% fat-free milk at room temperature for 2 h to block non-specific binding, the membranes were incubated with primary antibodies at appropriate dilutions in TBS-Tween (TBST; 0.01% Tween-20 in TBS) overnight at 4 °C. The membranes were then washed three times for more than 30 min with TBST and incubated with a secondary antibody (1:4,000 dilution) for 1 h at room temperature. The membranes were washed three times with TBST and visualized by enhanced chemiluminescence using the Immobilon Western Chemiluminescent HRP Substrate (Merck Millipore). Primary antibodies for RPL23 (16086-1-AP, 1:400) and p21^Cip1^ (10355-1-AP, 1:1000) were purchased from ProteinTech (USA). Miz-1 (sc-22837, 1:200) and p15^Ink4b^ (sc-612, 1:200) were purchased from Santa Cruz Biotechnology (USA), and c-Myc (5605, 1:1000), PIK3CG (5405, 1:1000), AKT (3063, 1:1000), p-AKT (Ser-473) (4060, 1:1000), pro-caspase3 (9665, 1:1000), cleaved caspase3 (9664, 1:1000) and GAPDH (2118, 1:1000) antibodies were purchased from Cell Signaling Technology (USA). p53 (AB21090-1, 1:500) was purchased from AbSci Technology (USA). Secondary goat-anti-rabbit antibody (HA1001, 1:10000) was purchased from HuaAn Biotechnology (China).

### BM trephine biopsy and immunohistochemistry

BM trephine biopsy (BMTB) was performed by experienced pathologists, and BM trephines were fixed in 4% paraformaldehyde, decalcified with 5% EDTA and embedded in paraffin blocks. Briefly, 4-μm-thick paraffin-embedded specimens were deparaffinized, hydrated, heated for antigen retrieval and incubated in 3% H_2_O_2_ to block endogenous peroxidase activity. The slides were treated with goat serum to prevent non-specific binding. The following primary antibodies were used: a mouse mAb against c-Myc (5605, CST, 1:800; cytoplasmic staining), a rabbit pAb against Miz-1 (sc-22837, Santa Cruz, 1:50; cytoplasmic staining) and a rabbit pAb against RPL23 (16086-1-AP, ProteinTech, 1:50; cytoplasmic staining). A mouse/rabbit immunohistochemistry (Enhanced Polymer) kit (PV-9000-D; ZSGB-BIO, China) was used to detect the primary antibodies. The sections were stained with a DAB kit. Counterstaining was performed with haematoxylin. Images of the slides stained for the target proteins were scanned at 40× magnification using an optical microscope (Olympus Co., Tokyo, Japan). We evaluated the staining of representative fields using Image-Pro Plus v6.0 software (Media Cybernetics, USA). After background removal via optical density calibration, the segmentation option was used to capture the areas of interest (AOIs) in each image with the HSI colour model parameter settings (H:0-30, S:0-255, and I:0-230), which covered the majority of the DAB-positive areas. The integrated optical densities (IODs) and positively stained regions in each image were measured and integrated as the IOD (sum) and the area (sum). The intensity of target protein expression was measured as the density mean = IOD (sum)/area (sum) (optical density/mm^2^ of AOI).

### Statistical analysis

The statistical analyses were performed using the SPSS software package (version 19, IBM SPSS), and all of the graphs were prepared using GraphPad Prism (version 5). All western blotting stripes were analysed based on a relative grey scale using Image-Pro Plus v6.0 software (Media Cybernetics, USA). Student’s t test was applied, and one-way ANOVA with post-hoc Tukey’s multiple comparison was performed for comparison of more than two groups. A significance cut-off of *p* < 0.05 was applied to all statistical tests. All data are expressed as the means ± S.E.M from three independent assays. The relationships between continuous variables were examined by calculating Pearson’s correlation coefficient. The *p* values are presented in the figures as follows: *p < 0.05, **p < 0.01, and ***p < 0.001.

## Electronic supplementary material

Supplementary Information
