# Supplementary material for: Ribosomal protein L23 negatively regulates cellular apoptosis via the RPL23/Miz-1/c-Myc circuit in higher-risk myelodysplastic syndrome
Source: Sci Rep. 2017 May 24;7:2323. doi: 10.1038/s41598-017-02403-x (PMC5443795; doi:10.1038/s41598-017-02403-x)

**Ribosomal protein L23 negatively regulates cellular apoptosis via the RPL23/Miz-1/c-Myc circuit in higher-risk myelodysplastic syndrome**

Yuekun Qi, Xiao Li, Chunkang Chang, Feng Xu, Qi He, Youshan Zhao, Lingyun Wu*

Department of Hematology, Shanghai Jiao Tong University Affiliated Sixth People’s Hospital

*Correspondence to: Lingyun Wu, MD, PhD, Dept. of Hematology, Shanghai Jiao Tong University Affiliated Sixth Hospital, Shanghai, China, 200233.

E-mail: lincy2032@163.com, Tel: +86-021-24058745, Fax: +86-021-64701361

**SUPPLEMENTARY INFORMATION**

**Supplementary Materials and Methods**

**SA-β-Gal assay**

β-Galactosidase staining of RPL23-NC/KD and wild-type SKM-1/K562 cells was performed with a senescence-associated β-Galactosidase Staining Kit (Beyotime, China). SKM-1 and K562 cells infection and culture are described in Methods of our manuscript, seventy-two hours after efficient transfection, cells were collected and washed three times with PBS and fixed with 4% paraformaldehyde for fifteen minutes at room temperature. Next, washed cells were incubated overnight at 37℃ in CO_2_ free incubator with the working solution containing 0.05 mg/ml 5-bromo-4-chloro-3-indolyl-b-d-galactopyranoside (X-gal)). The stained cells were planked into 6-well plates and scanned at 100× magnification using an optical microscope (Olympus Co., Tokyo, Japan). And the cell in blue was considered as positive for SA-β-gal staining. Percentages of SA-gal-positive cells were determined by scoring 300 cells, in triplicate, in three independent experiments.

**Superinfection of SKM-1 cells with LV-RPL23-RNAi and CMV-c-Myc expression plasmid vectors and control vectors**

Transfection of lentiviral RNAi vectors was described in Methods. Briefly, SKM-1 cells were seeded into 6-well plates (1x10^5^/well) and cultured for twenty-four hours to reach 80% confluence. lentiviral RNAi particles, negative control vectors and serum-free medium were added into 6-well plates at a MOI of 10 with 5 mg/ml Polybrene according to the following 5 groups: (a) WT, serum-free medium (used as blank control); (b) NC, LV-RPL23-NC particles (used as negative control); (c) RPL23-KD, LV-RPL23-RNAi particles; (d) RPL23-KD/c-Myc-OE, LV-RPL23-RNAi particles; and (e) c-Myc-OE, LV-RPL23-NC particles. Twelve hours later, the medium was replaced with CM and continued to culture for twenty-four hours. For induced expression of c-Myc, transfected SKM-1 cells were replaced with 1600μl serum-free RPMI-1640 medium and relevant transfection mixture (tube a: 200μl serum-free RPMI-1640 medium + 5μl lipo 2000; tube b: 200μl serum-free RPMI-1640 medium + 4μg relevant plasmids, mix two tubes well and rest for twenty minutes at room temperature before use) was added into specific wells: (a) WT, 400μl serum-free medium; (b) NC, transfection mixture with CMV-c-Myc-NC; (c) RPL23-KD, transfection mixture with CMV-c-Myc-NC; (d) RPL23-KD/c-Myc-OE, transfection mixture with CMV-c-Myc expression plasmids and (e) c-Myc-OE, transfection mixture with CMV-c-Myc expression plasmids. After six hours of incubation, medium was replaced with CM.

**Supplementary Table and Figure Legends**

**Supplementary Table S1. Characteristics of 97 MDS patients for qRT-PCR analysis.** RA, Refractory Anemia; RARS, Refractory Anemia with Ring Sideroblasts; RCMD, Refractory Cytopenia with Multilineage Dysplasia; RAEB-1, Refractory Anemia with Excess Blasts 1; RAEB-2, Refractory Anemia with Excess Blasts 2; Del(5q), 5q- syndrome. Low, Low-risk; Int-1/Int-2, Intermediate-1/Intermediate-2; High, High-risk.

**Supplementary Table S2. Primer sets used in this research for qRT-PCR.**

**Supplementary Fig. S1.** (a) The intrinsic mRNA expression levels of RPL23 in several cell lines. Knockdown efficiency of RPL23 was determined by qRT-PCR (b) and western blotting (c) in SKM-1 and K562 cell lines, and the histogram (d) denoted relative grey scale of RPL23 stripes normalized to GAPDH expression level using Image-Pro Plus v6.0 software (Media Cybernetics, USA). (e) The pro-apoptotic induction of RPL23 knockdown was evident based on a significant increase in an apoptosis marker (cleaved caspase-3) in RPL23-KD SKM-1 cells as determined by western blotting (first panel). Western blotting results did not reveal any alternations in p53 expression (e, second panel), and its downstream target gene of p53, HDM2, was not transcriptionally activated by qRT-PCR analysis. Target stripes were displayed as chopped. Original scans were provided in Supplementary Information (Supplementary Fig. S5). Each assay was performed in triplicate. Unpaired Student’s t tests were used to calculate all *p* values shown throughout the figure. Data was expressed as the means±S.E.M. WT: wild type; NC: RPL23-NC; KD: RPL23-KD. **p*<0.05; ***p*<0.01; ****p*<0.001.

**Supplementary Fig. S2.** qRT-PCR was used to measure superinfection efficiency of SKM-1 cells. RPL23-KD/c-Myc-OE group showed decreased expression of RPL23 (0.08±0.01, *p*<0.001) and the mRNA level of c-Myc was restored to 0.85±0.03 compared with the expression level in RPL23-KD group (0.58±0.08, *p*<0.05). Also, CMV-driven expression of c-Myc alone was convinced (1.87±0.20, *p*<0.01) compared with WT group. WT group was used for normalization to β-actin expression levels. Each assay was performed in triplicate. Unpaired Student’s t tests were used to calculate all *p* values shown throughout the figure. Data was expressed as the means±S.E.M. **p*<0.05; ***p*<0.01; ****p*<0.001.

**Supplementary Fig. S3.** Apoptotic analyses of SKM-1 cells superinfected with lentiviral and CMV-driven expression plasmid vectors. (a) On a GFP vs. side-scatter plot, GFP positive events (R1) were selected for transfection of LV-RPL23-RNAi-GFP expression vectors. (b) On a RFP vs. side-scatter plot, region, R2 represents RFP+ cells (gated by R1). (c) Annexin V-APC vs. side-scatter analysis of GFP+RFP+ cells gated by R1 and R2. Take NC group (co-infected with two negative control vectors) as an example. Apoptotic ratio was determined on annexin V-APC plots. The right quadrant represents the apoptotic cells, Annexin V-APC positive (R3).

**Supplementary Fig. S4.** Senescence-associated β-galaciosidase staining showed RPL23-NC/KD and wild type SKM-1 cells were negative for β-gal, while RPL23-NC/KD and wild type K562 cells showed moderate positive staining for senescent signals. However, percentages of SA-gal-positive cells didn’t show any statistical significance among the three groups in both cell lines (shown in the histogram). Clearly positively stained cells are indicated with arrows. Unpaired Student’s t tests were used to calculate all p values shown throughout the figure. Data was expressed as the means±S.E.M. WT: wild type; NC: RPL23-NC; KD: RPL23-KD; ND, not detectable. *p<0.05; **p<0.01; ***p<0.001.

**Supplementary Fig. S5.** Original Scans of all immunoblots depicted in the individual figures. The individual panels represent the full scans of the membranes cropped according to molecular weight of the target stripes and the marker shown in the individual panels.

**Supplementary Tables and Figures**

**Supplementary Table S1**

**Characteristics of 97 MDS patients for qRT-PCR and IHC analysis**

| Characteristics | Category |
| --- | --- |
| Median age (range) | 55 (18-85） |
| Sex, n (%) |  |
| Male | 55 (57） |
| Female | 42 (43） |
| WHO/FAB classification, n (%) |  |
| RA | 7 (7) |
| RARS | 8 (8) |
| RCMD | 34 (35) |
| RAEB-1 | 22 (23) |
| RAEB-2 | 20 (21) |
| Del(5q) | 6 (6) |
| International prognosis scoring system, n (%) |  |
| Low | 9 (9) |
| Int-1 | 45 (46) |
| Int-2 | 33 (34) |
| High | 10 (10) |

**Supplementary Table S2**

**Primer sets for qRT-PCR**

| Genes | Forward primer | Reverse primer |
| --- | --- | --- |
| β-actin | 5’- AATGTGGCCGAGGACTTTGATTGC -3’ | 5’- AGGATGGCAAGGGACTTCCTGTAA -3’ |
| RPL23 | 5’- GGTGTGAAGGTTGGCCTGAC -3’ | 5’- GGCACCTGGCTGACCATCAA -3’ |
| Miz-1 | 5’- CCGGCCTTTTGGAGATCTGA -3’ | 5’- CAAAGGTGCAGTCACAGAGG -3’ |
| c-Myc | 5’- TCCTCCCCACGGGCCAGCC -3’ | 5’- GGCAGGGGTTTGCCTCTTCT -3’ |
| p15^Ink4b^ | 5’- CCCTGCCACCCTTACCAGA -3’ | 5’- GCAGATACCTCGCAATGTCAC -3’ |
| p21^Cip1^ | 5’- CCACTTTGCCAGCAGAATAA -3’ | 5’- ACGGGACCGAAGAGACAAC -3’ |
| PIK3CG | 5’- GGCGAAACGCCCATCAAAAA -3’ | 5’- GACTCCCGTGCAGTCATCC -3’ |
| p53 | 5’- CAGCACATGACGGAGGTTGT -3’ | 5’- CATCCAAATACTCCACACGC -3’ |
| HDM2 | 5’- AACACTACTCAAGAGACCCTGG -3’ | 5’- GACGCAAACTGGTATCTTCACA -3’ |

**Supplementary Fig. S1.**


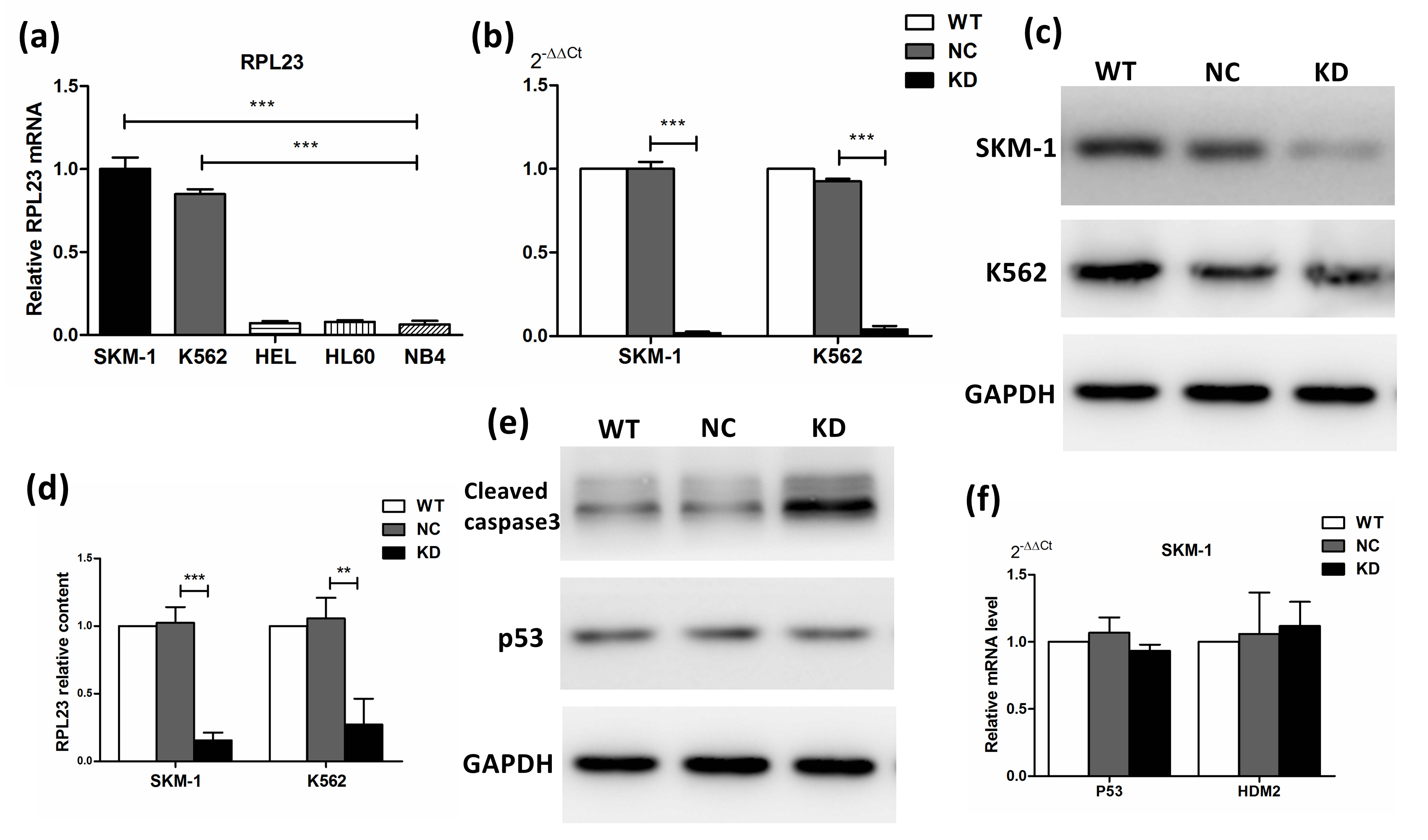


**Supplementary Fig. S2.**


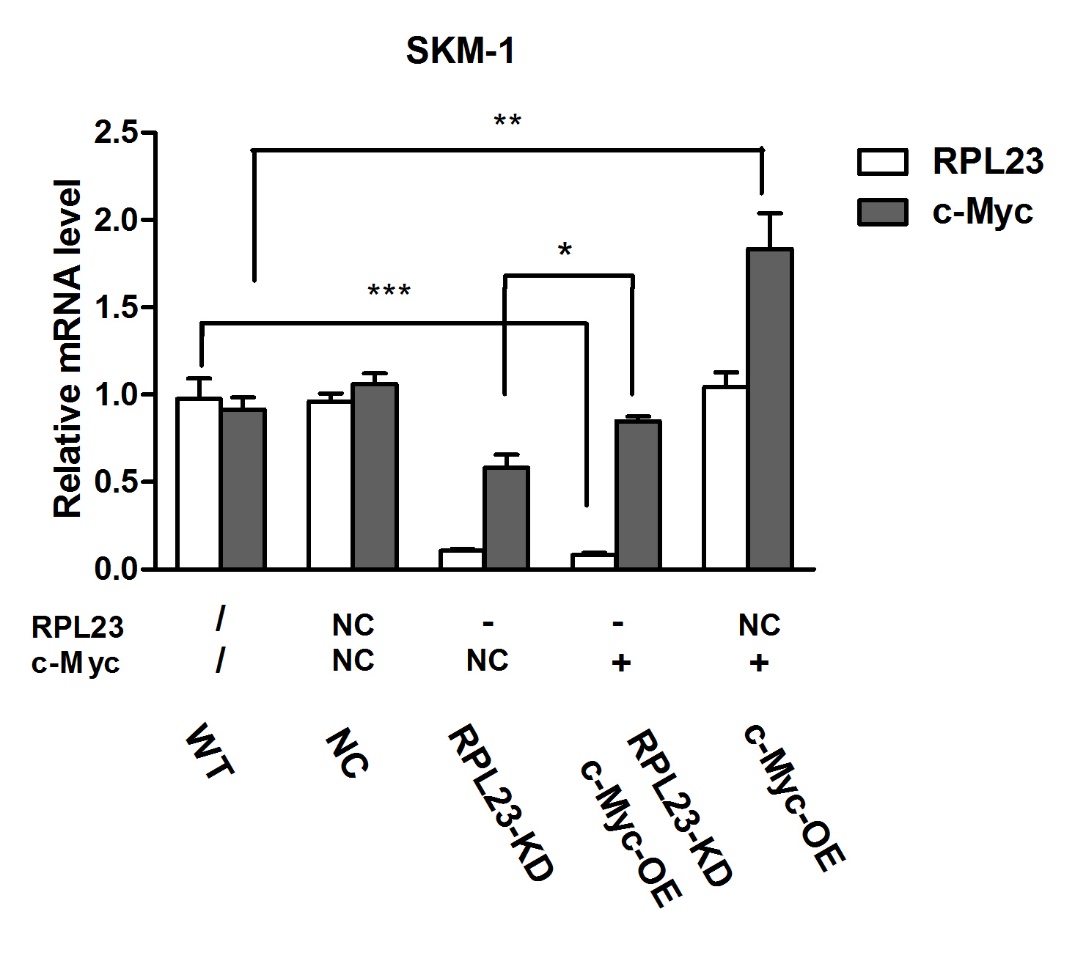


**Supplementary Fig. S3.**


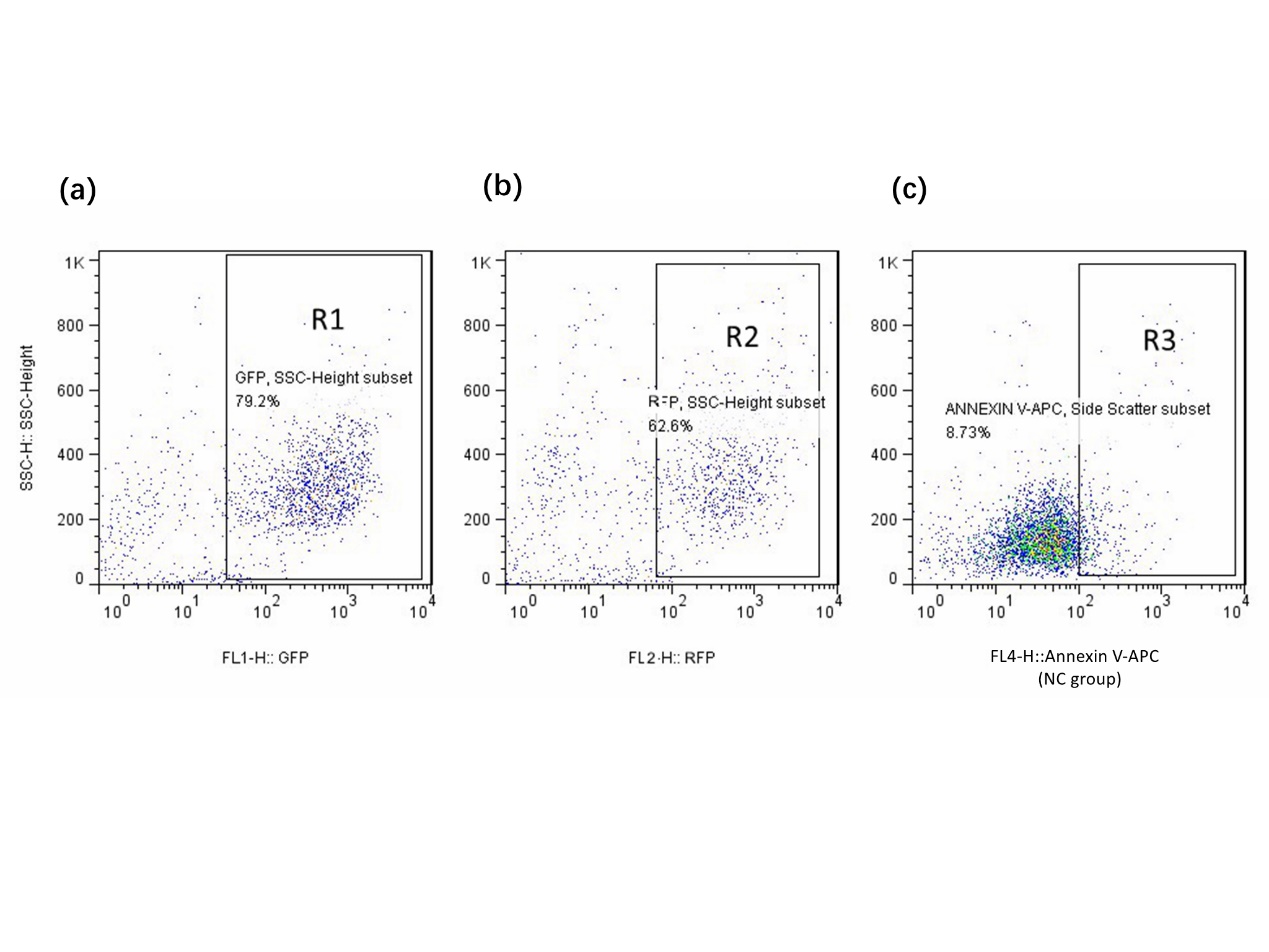


**Supplementary Fig. S4.**


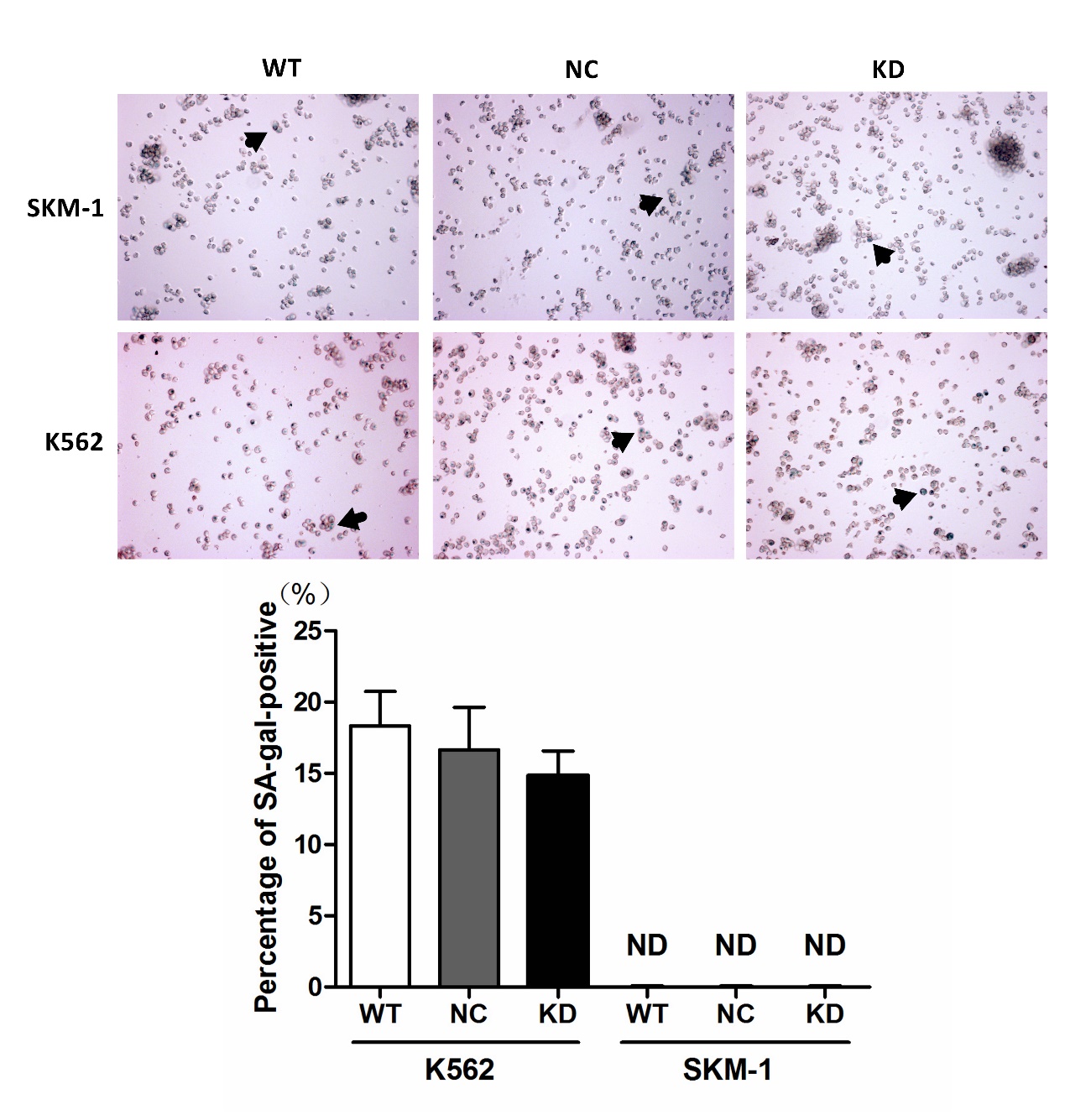


**Supplementary Fig. S5.**


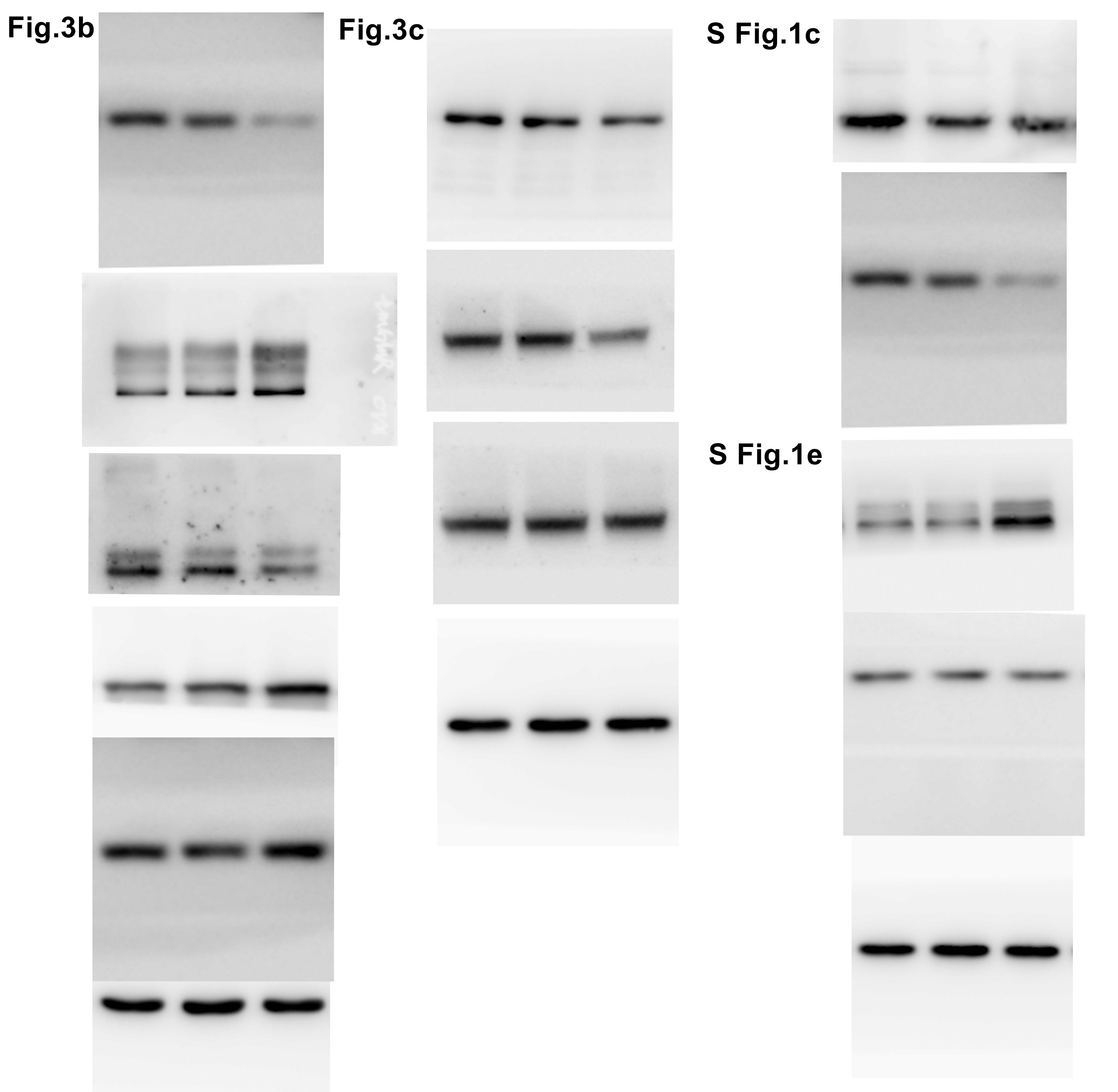

Supplement: Supplementary file 1 — Supplementary Information [file 41598_2017_2403_MOESM1_ESM.doc]
